# Breakthrough in 2D ultrahigh-*κ* dielectrics by paraelectric phase engineering in van der Waals crystal

**DOI:** 10.1093/nsr/nwag240

**Published:** 2026-05-01

**Authors:** Qinci Wu, Hailin Peng

**Affiliations:** Center for Nanochemistry, Beijing Science and Engineering Center for Nanocarbons, Beijing National Laboratory for Molecular Sciences, College of Chemistry and Molecular Engineering, Peking University, China; Center for Nanochemistry, Beijing Science and Engineering Center for Nanocarbons, Beijing National Laboratory for Molecular Sciences, College of Chemistry and Molecular Engineering, Peking University, China; Academy for Advanced Interdisciplinary Studies, Peking University, China

As semiconductor technology nodes aggressively scale down to the nanoscale regime, the integration of ultrathin gate dielectrics with ultrahigh dielectric constants ($\kappa $) has become imperative [1]. Concurrently, 2D semiconductors have emerged as a promising platform for sub-1-nm transistor architectures; their dangling-bond-free surfaces preserve high mobility at atomic thicknesses, thereby enabling rigorous electrostatic control, suppressing short-channel effects and minimizing passive power dissipation [2,3]. However, traditional deposited amorphous oxides suffer from dangling bonds and severe interface scattering, which critically degrade carrier mobility in 2D channels [4]. While van der Waals (vdW) materials intrinsically offer atomically flat, dangling-bond-free interfaces, the majority of known vdW insulators exhibit relatively low $\kappa $ values, severely limiting their application in advanced nanoelectronics.

**Figure 1.**
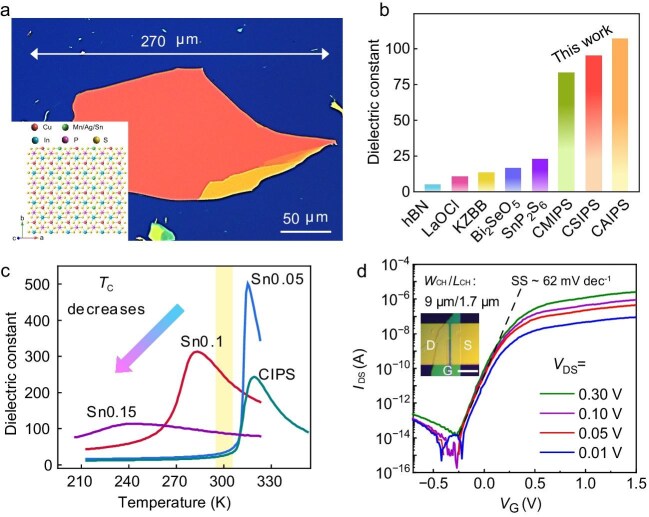
(a) Typical Ag-substituted CIPS (CAIPS) nanoflake exfoliated onto Si/SiO_2_ substrate. Inset: crystal structure of CM′IPS. (b) Comparison of the dielectric constant of typical vdW dielectric single crystals. (c) Dependence of dielectric constant on temperature for CIPS and Sn-substituted CIPS (CSIPS) bulk crystals. (d) Transfer characteristics (*I*_DS_−*V*_G_) of few-layer MoS_2_ field-effect transistor (FET). Inset shows the optical picture of the device. Adapted with permission from ref. [5].

A recent study has shattered this bottleneck by introducing a novel family of highly crystalline vdW dielectrics: substituted copper indium thiophosphate, Cu_1−_*_x_*M′*_x_*InP_2_S_6_ (CM′IPS, M′ = Sn, Ag, Mn; 0.05 ≤* x* ≤ 0.4). Through meticulous elemental substitution in CuInP_2_S_6_ (CIPS), Yang Chai and colleagues successfully synthesized centimeter-scale single crystals with uniform elemental distribution [5]. Furthermore, the intrinsic vdW layered architecture of CM′IPS enables its facile exfoliation into large-area, atomically smooth nanoflakes, achieving lateral dimensions up to 270 μm (Fig. 1a) and thicknesses down below 5 nm.

Comprehensive dielectric benchmarking demonstrates that the CM′IPS family achieves exceptional dielectric constants ($\kappa $) between 86 and 108, positioning these materials at the forefront of known vdW layered insulators (Fig. 1b). The underlying physical mechanism for this exceptionally high dielectric response is intrinsically linked to the stabilization of the paraelectric phase. This ferroelectric-to-paraelectric transition provides a universal mechanism for dielectric enhancement that operates independently of the specific electronic characteristics of the individual M′ substituents (Fig. 1c).

When implemented as gate dielectrics in logic devices, these ultrathin CM′IPS nanoflakes offer a pristine, trap-free vdW interface with 2D channel materials such as MoS_2_. This atomically sharp, scattering-free interface is critical for preserving uncompromised carrier mobility and maximizing gate electrostatic control in ultra-scaled devices. Consequently, the fabricated field-effect transistors deliver stellar performance metrics: they exhibit a formidable ON/OFF current ratio exceeding 10^8^, and achieve a nearly ideal subthreshold swing of 62 mV/dec (Fig. 1d). Building upon this exceptional single-device performance, the researchers successfully demonstrated the integrated-circuit potential of CM′IPS by fabricating multi-gate logic architectures. Ultimately, this work not only discovers a premier class of ultra-high-$\kappa $ vdW dielectrics but also establishes a robust pathway toward the realization of next-generation, high-performance 2D electronics.
